# Can Time-Lapse Incubation and Monitoring Be Beneficial to Assisted Reproduction Technology Outcomes? A Randomized Controlled Trial Using Day 3 Double Embryo Transfer

**DOI:** 10.3389/fphys.2021.794601

**Published:** 2022-01-04

**Authors:** Yu-han Guo, Yan Liu, Lin Qi, Wen-yan Song, Hai-xia Jin

**Affiliations:** ^1^Center for Reproductive Medicine, The First Affiliated Hospital of Zhengzhou University, Zhengzhou, China; ^2^Henan Key Laboratory of Reproduction and Genetics, The First Affiliated Hospital of Zhengzhou University, Zhengzhou, China; ^3^Henan Provincial Obstetrical and Gynecological Diseases (Reproductive Medicine) Clinical Research Center, The First Affiliated Hospital of Zhengzhou University, Zhengzhou, China; ^4^Henan Engineering Laboratory of Preimplantation Genetic Diagnosis and Screening, The First Affiliated Hospital of Zhengzhou University, Zhengzhou, China

**Keywords:** ART, time-lapse, embryo transfer, clinical outcomes, implantation

## Abstract

**Objective:** To determine if the application of time-lapse incubation and monitoring can be beneficial to clinical outcomes in assisted reproductive technology.

**Methods:** A total of 600 patients were equally randomized to three groups, namely, conventional embryo culture and standard morphological selection (CM group), time-lapse culture and standard morphological selection (TLM group), and time-lapse culture and morphokinetic selection (TLA group). Notably, 424 undergoing fresh autologous *in vitro* fertilization cycles were analyzed, 132 patients in the CM group, 158 in the TLM group, and 134 in the TLA group. Main outcomes included clinical outcomes, embryo development rates, and perinatal outcomes.

**Results:** Clinical pregnancy rates in the time-lapse groups were significantly higher than in the CM group (CM 65.2% vs. TLM 77.2% vs. TLA 81.3%). Implantation rates and live birth rates were significantly higher for the TLA group (59.7 and 70.9%) compared with the CM group (47.7 and 56.1%) but not compared with the TLM group (55.4 and 67.1%). There was no statistical difference in miscarriage and ectopic pregnancy rates among the three groups. Overall, birth weight was significantly higher in the time-lapse groups (CM 2,731.7 ± 644.8 g vs. TLM 3,066.5 ± 595.4 g vs. TLA 2,967.4 ± 590.0 g). The birth height of newborns in the TLM group was significantly longer than that of the CM group and TLA group (CM 48.3± 4.4 cm vs. TLM 49.8± 2.3 cm vs. TLA 48.5± 2.7 cm).

**Conclusion:** Time-lapse incubation and monitoring have a significant benefit on clinical pregnancy rates and on overall birth weights while morphokinetic analysis is not necessary.

**Clinical Trial Registration:** [www.ClinicalTrials.gov], identifier [NCT02974517].

## Introduction

One of the most important steps during assisted reproduction treatment is selecting the best embryo for transfer. Since the advent of assisted reproduction technology (ART), a conventional incubator with conventional morphological evaluation is most widely used to culture and select embryos, reaching a clinical pregnancy rate (CPR) in combination with conventional morphological evaluation of around 60% (Armstrong et al., 2018).

The application of the time-lapse system (TLS) for incubation and monitoring of human embryos in ART provides an alternative, which is non-invasive, and offers more embryo information. However, it has been debated whether using a TLS can improve the clinical outcome compared with conventional incubation.

Conventional incubation in combination with conventional morphological evaluation is subjective and requires almost daily embryo assessment, whereby exposing embryos to adverse temperature, humidity, and changes in the gas atmosphere may affect pH. On the contrary, TLS maintains stable culture conditions and provides kinetic parameters of embryos by continuous monitoring. A large retrospective cohort study (Meseguer et al., 2012) suggested that culturing and selecting embryos by TLS increases CPR. Other research reported high accuracy and specificity in predicting blastocyst progression by measuring early morphokinetic parameters (Wong et al., 2010; Petersen et al., 2016).

However, multiple randomized controlled trials (RCT) (Kirkegaard et al., 2012; Goodman et al., 2016; Barberet et al., 2018; Kovacs et al., 2019) reported no significantly improved implantation rate and CPR by TLS. In contrast, a meta-analysis of RCTs that used both effects of time-lapse, namely, undisturbed culture and additional information for embryo selection, reported a benefit in regard to ongoing pregnancy and live birth (Pribenszky et al., 2017, 2018). Still, there is a multitude of morphokinetic parameters that can be assessed by time-lapse and all require annotation to be performed by embryologists. A prospective study undertook a pairing analysis between published time-lapse algorithms and also compared the choice of embryologists (Storr et al., 2018). Only one pair showed very good agreement while 12 pairs showed poor agreement. The embryos were assessed as “best” by time-lapse algorithms, and the embryologist did not necessarily show good agreement either. Therefore, proper external validation is needed before time-lapse algorithms can be used in the clinic, especially if a clinic-specific algorithm should be adapted.

In recent years, the use of time-lapse incubation in combination with morphokinetic monitoring has increased and enabled creating more universal algorithms for embryo selection (Petersen et al., 2016) that may apply to *in vitro* fertilization (IVF) cases performed in different clinics. However, how this compares to standard morphology selection, either after conventional incubation or after time-lapse incubation, and how it affects clinical outcomes remains open. To address this, we conducted a prospective RCT that aimed to compare clinical outcomes of embryos selected by standard morphology in a conventional incubator or a time-lapse incubator compared with time-lapse incubation combined with selection by a universal morphokinetic scoring system. This study was further restricted to IVF with conventional insemination and not by intracytoplasmic sperm injection (ICSI).

## Materials and Methods

### Study Design and Participants

This single-center study was a three-arm, prospective RCT conducted from November 2016 to December 2019 at the First Affiliated Hospital of Zhengzhou University, China. Female patients were eligible for ≤ 37 years of age with normal ovarian reserve (FSH < 10 mIU/ml; antral follicle count > 5), undergoing their first fresh IVF cycle with conventional insemination using their oocytes due to tubal factor infertility without a history of hereditary diseases. Oocyte or sperm donation, ICSI, preimplantation genetic diagnosis, and womb diseases were the exclusion criteria. Only one cycle per patient was included. As most centers in China, our center currently performs most embryo transfers on Day 3, and most of the patients meet the criteria for double embryo transfer (DET). Usually, DET is feasible in combination with the preference of patients without scar uterus, uterine malformation, cervical insufficiency, short stature, and ovarian hyperstimulation syndrome. So, this study aimed at patients with DET on Day 3, and single embryo transfers (SETs) or blastocyst transfers were excluded.

### Randomization and Procedures

Randomization was performed immediately after oocyte denudation. All patients were randomly allocated *via* online-generated blocks^1^ to culture embryos in a conventional incubator (Minc, COOK, Australia) followed by conventional morphological evaluation by the same embryologist (Conventional-Manual Group, CM Group), in a time-lapse incubator (EmbryoScope^®^, Vitrolife, Sweden) combined with conventional morphological evaluations by the same embryologist [Time-Lapse-Manual Group, TLM Group] or in a time-lapse incubator with evaluation according to KIDScore Day 3 parameters (Petersen et al., 2016) (Time-Lapse-Auto Group, TLA Group). Due to the nature of study intervention, it was not possible to blind investigators to the embryo morphology assessments. However, for the analyses, the data manager, statistician, and embryologist were blinded to the allocation.

Institutional Review Board approval was obtained (no. 10/12/2016). The trial was registered at ClinicalTrials.gov (NCT02974517).

### Ovarian Stimulation and Insemination

Most of the patients in this study were treated by the management with early follicular phase long-acting gonadotropin-releasing hormone agonist (GnRH-a) long protocol: 3.75 mg of long-acting GnRH-a (Daphne, Beaufour Ipsen, France) was given for downregulation on Days 2–3 of menstruation. When the patient reached the downregulation standard after 30–42 days with no follicle > 10 mm in diameter, estradiol < 183 pmol/L, and luteinizing hormone (LH) < 3 IU/L, gonadotropin (Gn) was used for controlled ovarian hyperstimulation (COH) according to body weight, ovarian reserve, and ovarian response. When one dominant follicle diameter ≥ 20 mm, three follicles diameter ≥ 17 mm, or two-thirds follicles diameter ≥ 16 mm, 250 μg of ovidrel (Meker, Italy) and 2,000 IU of hCG (Livzon, China) were used. At 37 h after injection, oocyte retrieval was performed by the transvaginal ultrasound-guided follicle. After being identified under the stereomicroscope, the oocyte-corona-cumulus complex (OCCC) was transferred to G-MOPS Plus (Vitrolife, Sweden). After washing with G-IVF Plus (Vitrolife, Sweden) three times, the OCCC was transferred into G-IVF Plus medium and cultured in 37°C, 6% CO_2_, and 5% O_2_ incubators for insemination.

### Embryo Culture and Time-Lapse Recording

For IVF patients, 39–40 h after hCG injection, the semen after density gradient centrifugation and upstream was added into 50 μl G-IVF Plus micro drop, and the final sperm concentration reached approximately 10,000 sperm per oocyte. After 5 h of sperm and oocyte coculture, the cumulus cells around the oocytes were mechanically removed by the denudation pipette, and the second polar body extrusion was observed and recorded. Zygotes with two polar bodies were transferred into the time-lapse culture dish (EmbryoSlide, Vitrolife, Sweden). Each embryo was cultured in 25 μl of G1 Plus (Vitrolife, Sweden) overlaid with mineral oil and incubated at 37°C and 6% CO_2_, 5% O_2_, and 89% N_2_ in the conventional or time-lapse incubator, respectively. The gas mixing of conventional incubator and time-lapse incubator is the same without any difference. For conventional incubator, the embryos were observed out of the incubator 4 times: 16–18 h after insemination (observation of pronucleus), 26–28 h after insemination (observation of early cleavage), 44–46 h after insemination, and 66–68 h after insemination (observation of cleavage embryos). In the time-lapse incubator, images of each embryo were acquired every 10 min at seven focal planes. After observing the embryo morphology in the cleavage stage on Day 3, the embryos were arranged for cryopreservation, transferred on Day 3, cultured for blastocyst progression, or discarded. Embryos arranged for blastocyst cultured were placed in a conventional incubator and cryopreserved on Day 5. Day 3 cleavage-stage embryos were used for the transfer, which was performed using a Wallace catheter (Mexico) under ultrasonographic guidance.

The embryos of patients included in this study were placed in a separate conventional incubator and separately from other patients. Therefore, observing the embryos of other patients would not bring additional gas condition changes and light exposure to the embryos in our study.

### Embryo Scoring and Selection

Standard/conventional embryo scoring in the CM Group and TLM Group was performed on Day 3 according to published standards (Brinsden, 2005). In short, cleavage-stage embryos were classified into 4 grades: Grade I: the blastomeres were in equal size and regular shape with intact zona pellucida; the cytoplasm did not contain granules; and the fragmentation is less than 10%. Grade II: the blastomeres were slight in unequal size and non-regular shape; the cytoplasm contained granules; and the fragmentation is less than 20%. Grade III: the blastomeres were in unequal size and non-regular shape; the cytoplasm contained evident granules; and the fragmentation is less than 50%. Grade IV: the blastomeres were in severely unequal size and non-regular shape; the cytoplasm contained severely evident granules; and the fragmentation is more than 50%. Grade I and II embryos were considered as top-quality embryos. Morphological scores were also used in the TLA group just for comparing with the other groups but the morphological scores were not used for embryo selection. In the TLA group, morphokinetic time-lapse parameters were used for embryo assessment and selection according to the KIDScore Day 3 algorithm, details of which are described in the initial study of Petersen et al. (2016). In short, embryos were classified into five categories according to KIDScore, and the two embryos with the highest score in a cohort of embryos from a patient were selected for DET. Embryos with abnormal cleavages, such as reverse cleavage or direct cleavage from zygote to > 2 cells, fall into the low KIDScore categories and were discarded.

### Outcome Measures and Endpoints

The primary endpoint for this study was CPR, which was calculated by dividing the number of clinical pregnancies by the number of patients with DET. The gestational sac was detected by ultrasound 35 days after transfer to diagnose clinical pregnancy. We analyzed fertilization rates, implantation rates, percentage of miscarriages, delivery rates, live birth rates, and the birth weight and birth height of the newborn for secondary endpoints.

### Statistical Analysis

#### Sample Size Calculation

This study was a superiority trial, and the sample size was calculated based on the CPR. According to the previous data in our center, the CPR of a conventional incubator with morphological evaluation is approximately 65%. To detect a 10% increase in implantation rate at 0.05 alpha risk and 80% power, we need to randomly select 399 subjects, 133 in each group. We increased the sample size to 600 patients in total to compensate for potential dropouts.

#### Analyzed Population

The analyzed population excluded SET cycles and blastocyst transfer cycles.

All analyses were conducted using SPSS Statistics 26.0 (IBM, United States). Continuous variables were presented as mean ± SD and compared using the *t*-test or one-way ANOVA test. Categorical variables were presented as *n* (%) and compared using the Chi-square test and Fisher’s exact test. *P* < 0.05 was considered statistically significant, and the multiple comparisons were corrected using the Bonferroni test for *P*-value.

## Results

**FIGURE 1:**
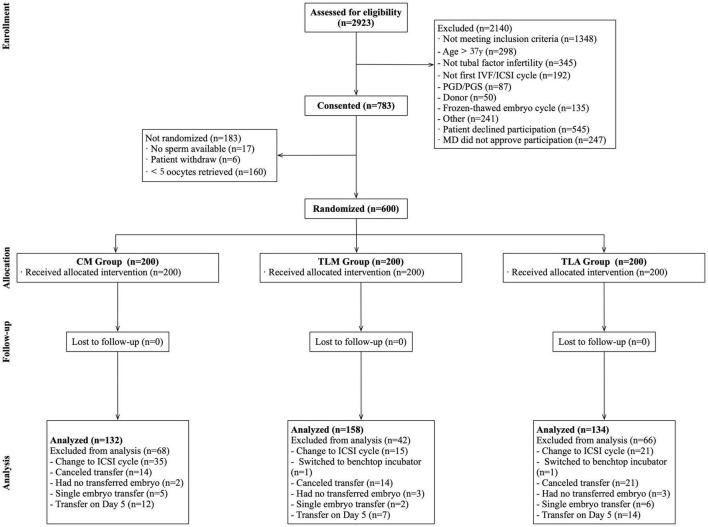
Study flowchart. IVF, *in vitro* fertilization; ICSI, intracytoplasmic sperm injection; PGD/PGS, preimplantation genetic diagnosis/screening; MD, Doctor of Medicine.

A total of 600 patients were randomized (study flowchart is shown in Figure 1) and 200 were allocated into each group. A total of 176 patients were excluded from the analysis (CM: 68; TLM: 42; TLA: 66), mostly due to a switch to ICSI, cancelation of the embryo transfer, and switch to culture to Day 5 or SET. The final number of patients who completed the procedure was 132 in the CM group (conventional incubator with conventional morphological evaluation), 158 in the TLM group (time-lapse incubator with conventional morphological evaluation), and 134 in the TLA group (time-lapse incubator with KIDScore), whereby reaching the anticipated number of 133 patients per group.

Demographic and cycle characteristics for the three groups are shown in Table 1. Mean age, body mass index (BMI), basal anti-Mullerian hormone (AMH), duration of infertility, and a number of retrieved oocytes were similar among the groups. The primary infertility rate was significantly higher for the TLM group (62.4%) compared with the TLA group (41.4%). Since all the patients included were tubal factor infertility, primary infertility or secondary infertility can only explain whether the patient had been pregnant before. Therefore, the differences in infertility types have no effect on intra-group comparability and endpoints.

**TABLE 1:** Demographic and cycle characteristics for the procedure-completed patients in three groups.

Variable	CM group (*n* = 132)	TLM group (*n* = 158)	TLA group (*n* = 134)	TLM+TLA group (*n* = 292)	*P*_1_	*P*_2_	*P*_3_
Age (y)	29.2 ± 3.5	29.1 ± 3.1	29.6 ± 3.4	29.3 ± 3.2	0.689	0.195	0.406
BMI (kg/m^2^)	22.7 ± 3.2	22.8 ± 3.2	22.9 ± 3.2	22.9 ± 3.2	0.533	0.820	0.803
Primary infertility (%)	79/132 (59.8)	98/157 (62.4)	55/133 (41.4)	153/290 (52.8)	0.175	<0.001	0.001*^a^^,^^b^*
Secondary infertility (%)	53/132 (40.2)	59/157 (37.6)	78/133 (58.6)	137/290 (47.2)			
Length of infertility (m)	36.3 ± 28.7	40.1 ± 26.0	38.5 ± 34.1	39.4 ± 29.9	0.318	0.665	0.550
Baseline AMH (mIU/mL)	3.5 ± 3.1	4.0 ± 3.0	4.1 ± 3.0	4.0 ± 3.0	0.114	0.772	0.276
No. of retrieved oocytes	11.6 ± 2.8	11.1 ± 2.7	11.4 ± 2.7	11.2 ± 2.7	0.168	0.393	0.270

*n, number of patients; CM Group, tri-gas incubator with conventional morphological evaluation; TLM Group, time-lapse incubator with conventional morphological evaluations; TLA Group, time-lapse incubator with KIDScore;*

*P_1_, comparison of CM Group vs. TLM+TLA Group with t-test and Chi-square test;*

*P_2_, comparison of TLM Group vs. TLA Group with t-test and Chi-square test;*

*P_3_, comparison of CM Group vs. TLM Group vs. TLA Group with ANOVA and Chi-square test;*

*^a^Significance is referred to the comparison of CM Group vs. TLA Group;*

*^b^Significance is referred to the comparison of TLM Group vs. TLA Group.*

### Embryo Development Rates

The rates of normal fertilization and 2PN cleavage were equivalent among the different groups (Table 2). There was no difference in the percentage of top-quality embryos (Grade I + II embryos) among the three groups for Day 3, where the top-quality score within each group was determined by the same morphological criteria according to a published standard (Brinsden, 2005).

**TABLE 2:** Embryo development rates for the as-treated patients in three groups.

Variable	CM group	TLM group	TLA group	TLM + TLA group	*P*_1_	*P*_2_	*P*_3_
Normal fertilization (%)	921/1,535 (60.0)	1,070/1,755 (61.0)	941/1,525 (61.7)	2,011/3,280(61.3)	0.385	0.666	0.625
No. of viable embryos	4.5 ± 2.0	4.6 ± 2.0	4.8 ± 1.9	4.7 ± 2.0	0.431	0.482	0.572
2PN cleavage (%)	911/921 (98.9)	1,058/1,070 (98.9)	930/941(98.8)	1,988/2,011(98.9)	0.890	0.920	0.986
Top-quality embryo (%)	629/911 (69.0)	738/1,058 (69.8)	679/930(73.0)	1,417/1,988(71.3)	0.221	0.109	0.134

*CM group, tri-gas incubator with conventional morphological evaluation;*

*TLM group, time-lapse incubator with conventional morphological evaluations;*

*TLA group, time-lapse incubator with KIDScore;*

*P_1_, comparison of CM group vs. TLM + TLA group with t-test and chi-square test;*

*P_2_, comparison of TLM group vs. TLA group with t-test and chi-square test;*

*P_3_, comparison of CM group vs. TLM group vs. TLA group with ANOVA and chi-square test;*

*Top-quality embryo, Grade I and II embryos according to published standards (Brinsden, 2005).*

### Pregnancy Outcomes and Perinatal Outcomes

Table 3 shows the pregnancy outcomes and perinatal outcomes in each group. The CPR of the TLA group and the TLM group was significantly higher than that of the CM group (CM 65.2% vs. TLM 77.8% vs. TLA 81.3%; *P* = 0.006). The TLA group showed the highest implantation rate compared with the other groups (CM 47.7% vs. TLM 55.4% vs. TLA 59.7%), which was significantly different to the CM group (*P* = 0.019) but not to the TLM group, whereas TL (TLM and TLA combined) on its own (57.4%) was significantly different to CM (*P* = 0.009). A similar result was observed for the live birth rate (CM 55.3% vs. TLM 63.9% vs. TLA 70.1%; *P* = 0.042 and *P* = 0.023 for TLM + TLA 66.8% vs. CM). Miscarriage rate showed no statistical difference as well as ectopic pregnancy rate. The overall birth weight of newborns was significantly higher in TLM and TLA groups than in the CM group [CM group 2,731.7 ± 644.8 g vs. TLA group 3,066.5 ± 595.4 g vs. TLM group 2,967.4 ± 590.0 g (*P* < 0.001)]. The birth height of newborns in the TLM group was significantly longer than that of the other groups [CM group 48.3 ± 4.4 cm vs. TLM group 49.8 ± 2.3 cm vs. TLA group 48.5 ± 2.7 cm (*P* = 0.007)].

**TABLE 3:** Clinical outcomes and perinatal outcomes for the procedure-completion patients in three groups.

Variable	CM group	TLM group	TLA group	TLM + TLA group	*P*_1_	*P*_2_	*P*_3_
Clinical pregnancy (%)	86/132 (65.2)	123/158 (77.8)	109/134 (81.3)	232/292(79.5)	0.002	0.461	0.006^a^^,^^b^
Implantation (%)	126/264 (47.7)	175/316 (55.4)	160/268 (59.7)	335/584(57.4)	0.009	0.293	0.019^b^
Miscarriage (%)	11/86 (12.8)	16/123 (13.0)	12/109 (11.0)	28/232(12.1)	0.862	0.641	0.885
Ectopic pregnancy (%)	0/86 (0)	2/123 (1.6)	3/109 (2.8)	5/232(2.2)	0.387	0.555	0.308
Abnormal fetal (%)	1/86 (1.2)	4/123 (3.3)	0/109 (0)	4/232(1.7)	1.000	0.058	0.130
Live birth (%)	73/132 (55.3)	101/158 (63.9)	94/134 (70.1)	195/292(66.8)	0.023	0.260	0.042^b^
Gestational week	37.3 ± 2.5	38.1 ± 1.8	37.8 ± 1.9	38.0 ± 1.8	0.028	0.268	0.054
Singleton	38.3 ± 2.3	38.8 ± 1.0	38.8 ± 1.3	38.6 ± 1.5	0.267	0.750	0.152
Twin	36.2 ± 2.2	36.4 ± 1.9	36.0 ± 1.4	36.6	0.402	0.362	0.713
Weight of newborn (g)	2731.7 ± 644.8	3066.5 ± 595.4	2967.4 ± 590.0	3018.3 ± 593.7	<0.001	0.180	<0.001^a^^,^^b^
Singleton	3235.3 ± 563.2 (*n* = 41)	3426.7 ± 462.2 (*n* = 75)	3389.8 ± 428.1 (*n* = 63)	3409.9 ± 445.8 (*n* = 138)	0.043	0.635	0.117
Twin	2426.5 ± 480.4 (*n* = 66)	2628.2 ± 419.8 (*n* = 62)	2570.9 ± 422.1 (*n* = 64)	2598.4 ± 420.3 (*n* = 126)	0.011	0.449	0.032^a^
Height of newborn (cm)	48.3 ± 4.4	49.8 ± 2.3	48.5 ± 2.7	49.3 ± 2.5	0.127	0.003	0.007^a^^,^^c^

*CM group, tri-gas incubator with conventional morphological evaluation;*

*TLM group, time-lapse incubator with conventional morphological evaluations;*

*TLA group, time-lapse incubator with KID scoring;*

*P_1_, comparison of CM group vs. TLM + TLA group with t-test and chi-square test;*

*P_2_, comparison of TLM group vs. TLA group with t-test and chi-square test;*

*P_3_, comparison of CM group vs. TLM group vs. TLA group with ANOVA and chi-square test.*

*^a^Significance is referred to the comparison of CM group vs. TLM group.*

*^b^Significance is referred to the comparison of CM group vs. TLA group.*

*^c^Significance is referred to the comparison of TLM group vs. TLA group.*

*n, number of newborns.*

## Discussion

In the physiological state, embryos grow in a light-protected environment *in vivo* with constant temperature and humidity and at low oxygen. For embryos cultured *in vitro*, a poor *in vitro* culture environment may affect the outcome of embryo development and even have long-term effects on the safety of offspring. To simulate the culture environment of embryos *in vivo*, the excellent and stable performance of incubators is essential for *in vitro* embryo culture. Most centers currently apply conventional incubators, which can provide ideal temperature, humidity, and gas/pH conditions for gametes and embryos (Swain, 2014). However, the need for frequent incubator openings for assessing the developmental and morphological status of the growing embryos over time leads to fluctuations in temperature, humidity, gas, and pH and constitutes a constant stress factor for the developing embryos. As a result, gamete and embryo development *in vitro* is potentially compromised, which is an unavoidable weakness of conventional incubators (Mizobe et al., 2014). The introduction of time-lapse incubation systems enabled embryo observation and scoring without the need to remove dishes holding embryos from the incubator, whereby maintaining a stable culture environment at all times of development (Magdi et al., 2019). However, the light exposure of TLS may be harmful to the embryos (Herrero and Meseguer, 2013).

Many studies have reported that the use of time-lapse incubators improves embryo development and clinical outcomes (Barberet et al., 2018; Sciorio et al., 2018; Kovacs et al., 2019; Mascarenhas et al., 2019). However, whether this is due to the strictly controlled and undisturbed culture environment provided by time-lapse incubators or to better embryo selection based on the ability to assess morphokinetic parameters is still under debate and requires further prospective RCT. To contribute to this, we conducted this trial to compare embryo development and clinical outcomes among different incubation conditions and in regard to different strategies for embryo selection. In particular, we compared conventional incubation and selection by standard morphology scoring parameters (Brinsden, 2005) to time-lapse incubation either applying the same morphological scoring parameters or by using a universal algorithm based on morphokinetic parameters (Petersen et al., 2016).

It is important to note that the current standard in most IVF clinics in China is to perform a DET on Day 3; and that the mean female patient age is lower compared with most Western countries. Therefore, by design, this study may differ from many other studies in the field but has a clinical relevance due to the high cycle number, which is in general performed with DET on Day 3 in China as well as in other countries, especially in Asia.

Our results using Day 3 DET from conventional vs. time-lapse incubation show significantly higher implantation, clinical pregnancy, and live birth rates in time-lapse incubation, as already reported by others (Rubio et al., 2014) and summarized in a meta-analysis (Pribenszky et al., 2017, 2018). Whether embryos were selected after time-lapse incubation by morphological or by morphokinetic parameters did not make a significant difference. This may be an effect of incubation to Day 3 only and using always two embryos for transfer, meaning that this strategy somehow masks the full potential of time-lapse incubation and selection by an algorithm, which may only be visible by SET at the blastocyst stage (Ueno et al., 2021).

Looking at neonatal outcomes and in particular, at birth weights, these were in general higher in the time-lapse groups compared with conventional incubation, and these results were the same for the subgroup analysis of singleton and twin births. Looking at the recently published growth standards for newborns in China (Zong and Li, 2020), the birth weight for singletons born from TL incubation lies in the P50–P90 range with a mean gestational age of 38.8, whereas this is in the P25–P75 range for conventional incubation in combination with a shorter mean gestational age of 38.3. The observed differences in birth weights between newborns from conventional incubation compared with time-lapse incubation are somehow similar to those reported by others (Insua et al., 2017; Mascarenhas et al., 2019). However, some publications reported no difference in birth weights (Kovacs et al., 2019). In particular, a recent study from China compared conventional incubation with time-lapse incubation and reported neonatal outcome data (Ma et al., 2021). The authors found higher clinical pregnancy and implantation rates in the TLS but no differences in neonatal outcomes. This study differed from our study as it included IVF and ICSI, cleavage stage and blastocyst stage transfer, SET, and DET, and the strategy for embryo selection in the TLS group was not specified. Therefore, the two studies cannot be compared, but the overall benefit by using TLS was also shown in the study by Ma et al. (2021). In our study, the twin newborn birth weight of conventional incubator (2,426.5 ± 480.4 g) is lower than the normal level (2,500–4,000 g), which means that they are small for gestational age (SGA). The application of time-lapse can significantly increase the birth weight of newborns (*P* = 0.011) to normal weight. To maintain body temperature, SGA will consume more brown adipose tissues, which may lead to hypoglycemia, hypoxia, and even acidosis. In addition, SGA is more prone to respiratory symptoms (McGuire, 2017). The application of TLS may help to improve this problem, but further research is needed.

During early embryogenesis, there are two epigenetic reprogramming, including DNA methylation and histone modification of chromatin, resulting in changes in gene expression and phenotypic characteristics (von Wolff and Haaf, 2020). This process is vulnerable to external factors. Studies have found that there are differences in DNA methylation of Grb10 between placenta conceived through ART and placenta conceived naturally (Turan et al., 2010). Grb10 is an imprinted gene that regulates fetal and placental growth. Grb10 levels are elevated in fetuses with growth restriction (Sullivan-Pyke et al., 2017). These phenomena suggest that ART technology may lead to epigenetic changes and may change fetal phenotype and long-term health status.

Overall, these results are consistent with the view that a non-invasive culture environment can contribute to better clinical outcomes in regard to pregnancy, implantation, and live birth rates. Some studies suggest that time-lapse incubation may improve *in vitro* development of human embryos especially during early cleavage stages (Sciorio et al., 2018). However, our results do not support this view.

Embryo development is a procedural process, which means that essential events occur at fixed time points. The most common means to assess an embryo is morphological assessment under the light microscope, requiring to observe embryos at these time points. Morphological assessment is easy to perform but is subjective and inaccurate (Fishel et al., 2020). Moreover, embryo development is a dynamic process. The morphology of the embryo may change significantly in just a few minutes, resulting in morphological score changes. Thus, conventional morphological assessment, which is not comprehensive enough, may overestimate or underestimate the developmental potential of an embryo. The application of time-lapse provides data that are not available with conventional embryo assessment methods. TLS allows for continuous dynamic observation of embryo development, recording information on various parameters during embryo development, which can be used to efficiently select the embryos with the best developmental potential for transfer (Meseguer et al., 2011; Goodman et al., 2016; Petersen et al., 2016). A preclinical validation study showed that the best embryos selected by different algorithms are different for the same patient embryos among the published time-lapse algorithm, and the agreement between the algorithm and selection of embryologists is also poor (Storr et al., 2018). KIDScore is a morphodynamic algorithm that predicts blastocyst development based on morphological and morphodynamic events to select embryos with higher scores for transfer or freezing. The predictive capacity of the KIDScore Day 3 exceeded that of other published algorithms that were present at that time (Petersen et al., 2016). Our results showed no significant differences in the implantation rate, CPR, live birth rate, birth height, and birth weight between time-lapse incubation and morphological or morphokinetic selection. This indicates that KIDScore Day 3 agrees well with the selection of embryologists in our clinical laboratory setting, which cannot be generalized to other laboratories.

It is tempting to speculate that a time-lapse incubation system using a fully automated algorithm, that excludes any annotation bias or subjectivity by the human factor, which is the embryologist, may give better results. However, this is currently under investigation, and a recent study showed that, for blastocyst transfer, live birth rates were not significantly different if embryos were selected either by embryologists using either KIDScore Day 5 or Gardner score, or by a fully automated embryo scoring system (iDAScore) (Ueno et al., 2021). Still, it must be acknowledged that a fully automated system that does not require any assessment or interaction by embryologists will substantially ease the workflow in the laboratory.

However, even though this trial was controlled and randomized, some limitations exist. Our study was powered on the previously observed implantation rate of 65% after conventional incubation with morphological evaluation and selection, and we expected a difference of 10% in the implantation rate by using TLS. In the RCT, the CM group showed an implantation rate of 47.7%, the TLM group of 55.4%, and the TLA group of 59.7%. A 10% difference was only reached for the TLA group, which was significantly different vs. the CM group, but not in the TLM group, although the underlying sample size of 133 patients in each group was reached. This may point to a benefit of using time-lapse incubation in combination with an algorithm rather than standard morphology on Day 3 in DET. However, results are only applicable to the culture system and culture environment of our center. Besides, all patients included in this study were patients with tubal factor infertility, and no ICSI cycles were included, for which our conclusion has certain limitations, too. Another limitation of this study is that the embryos of the CM group left the incubator 4 times before transfer, whereas there was no control group with embryos taken out for observation in the time-lapse group. Therefore, we cannot clarify whether the difference in the outcome was due to the design of the time-lapse incubator itself being superior to the conventional incubator or due to the change in the culture environment of the conventional incubation.

The application of TLS can improve the clinical pregnancy and neonatal outcome to a certain extent, while this technology costs more. The patients in our center try the TLS for free, and it has a high degree of acceptance. If there is a charge, doctors and patients should consider the cost-benefit factor to decide whether to choose time-lapse incubation to improve IVF outcomes.

## Conclusion

Our study demonstrates that time-lapse incubators can improve reproductive outcomes to some extent compared with conventional incubators. Using time-lapse incubation can be beneficial to improve pregnancy rate, live birth rate, and neonatal outcome. Morphokinetic analysis is slightly better than standard morphology evaluations, but it is not significant. Therefore, it is suggested that using the time-lapse incubator to culture embryos can potentially maximize the benefit for the patients. Time-lapse with KIDScore supports *in vitro* development of human embryos at least as good as other standard incubation methods and may improve clinical outcomes.

## Data Availability Statement

The raw data supporting the conclusions of this article will be made available by the authors, without undue reservation.

## Ethics Statement

The studies involving human participants were reviewed and approved by the Ethics Committee of Scientific Research and Clinical Trial of The First Affiliated Hospital of Zhengzhou University. The patients/participants provided their written informed consent to participate in this study.

## Author Contributions

H-XJ: study design, embryology, clinical intervention, data entry and collection, editing, and writing assistance. Y-HG: data analysis and interpretation, and manuscript writing. YL, LQ, and W-YS: embryology, clinical intervention, editing, and writing assistance. All authors contributed to the article and approved the submitted version.

## Conflict of Interest

The authors declare that the research was conducted in the absence of any commercial or financial relationships that could be construed as a potential conflict of interest.

## Publisher’s Note

All claims expressed in this article are solely those of the authors and do not necessarily represent those of their affiliated organizations, or those of the publisher, the editors and the reviewers. Any product that may be evaluated in this article, or claim that may be made by its manufacturer, is not guaranteed or endorsed by the publisher.

## Footnotes

1
www.random.org
